# Knowledge, Attitudes and Practices regarding complex generic drugs among physicians in India: A cross-sectional survey

**DOI:** 10.1186/s12889-026-28768-1

**Published:** 2026-07-30

**Authors:** Amatha Sreedevi, Saurabh Singh, Usha Rani, Virendra S Ligade

**Affiliations:** 1 Department of Pharmaceutical Regulatory Affairs and Management, Manipal College of Pharmaceutical Sciences, Manipal Academy of Higher Education, Manipal, India; 2 Department of Health Care and Hospital Management, Prasanna School of Public Health, Manipal Academy of Higher Education, Manipal, India

**Keywords:** Complex generics, Knowledge, Attitude, Practice, Government and private doctors

## Abstract

**Background:**

Complex generics are gaining importance as a affordable alternative to high cost complex drugs. However their successful adoption by end user largely depends in the hands of physicians understanding and confidence towards these products. Despite of the market growth of complex generics, evidence related to knowledge, attitude and practice (KAP) of physician towards complex generics remain limited particularly in India. Therefore the current study assessed KAP and perspectives related to regulatory and quality.

**Methods:**

An online cross sectional survey was conducted among 100 licensed physician practicing complex generic across 21 states of India. A validated and pilot tested 52 item questionnaire consisting of five domains: knowledge, attitude, practice, regulatory and quality perspective of complex generics were assessed and employed for collecting responses. Data were analyzed using descriptive and inferential parametric statistics such as Pearson’s correlation and multiple linear regression analysis in Jamovi software. *p*-value less than 0.05 were considered as statistically significant.

**Results:**

Most participants (65% of physicians) have shown moderate awareness about the product and merely 16% had an in-depth knowledge about what is meant by complex generics. Even identification of the products under the class of complex generics were correctly identified by only 2% respondents although they are prescribing these drugs. Approximately 49% expressed neutral confidence regarding the quality, safety, and efficacy of complex generics. More than 70% of the participants highlight the need for more stringent regulatory scrutiny and educational initiatives. Knowledge showed significant positive correlation with all the other four domains such as attitude, practice, regulatory and quality perspective with all p vale < 0.001.

**Conclusion:**

In the current exploratory survey study, the participants demonstrate positive attitude towards regulatory and academic initiatives, however moderate awareness and limited knowledge as well as confidence in prescribing complex generics. From the non-probability sampling approach, these findings concluded that limited access to information on scientific and regulatory data and also knowledge gaps has reduces physician confidence in prescribing these medicines. Strengthening academic foundation, awareness training activities, dissemination of regulatory and clinical evidence will help in improving prescribing confidence and market acceptance which ultimately support affordable healthcare access.

**Supplementary Information:**

The online version contains supplementary material available at https://doi.org/10.1186/s12889-026-28768-1.

## Background

The rising cost of healthcare is a grave problem for rapidly rising burden of chronic diseases has paved the way to affordable high-quality medicines worldwide [1]. Generic drugs play a significant role and provide significant benefits, including cost savings, increased accessibility, therapeutic equivalence, and trustworthy quality and safety [2]. However, not all generics are develop and evaluate equally. It depends on the target, route of administration, nature of API, formulation characteristics and several factors associated with establishing bioequivalence [3]. Complex generic products such as long-acting injectables, liposomal formulations metered-dose inhalers, transdermal patches, or drugs with complex APIs are significantly more difficult and expensive to develop due to unique challenges [4]. Unlike small molecules, complex generics require extensive analytical characterization to demonstrate pharmaceutical equivalence [5]. There is no specific regulatory procedure available for the approval of complex generics, unlike small molecule generics and biosimilars [6].

Globally, regulatory agencies such as the United States Food and Drug Administration (US FDA) and the European Medicines Agency also acknowledges the fact that more in-vivo investigations are needed for further research on the scientific and regulatory complexities and approval of complex generic or hybrid medicines [7]. Developing complex generics is very challenging, time-consuming, and expensive, and it also requires demonstrating the equivalency, safety, and efficacy of the therapy [8]. The FDA has come up with a pre- Abbreviated New Drug Application (pre-ANDA) program which offers the manufacturers scientific advice regarding the development of the product which encompasses recommendations of the study of bioequivalence and the general approach to clinical trials [9]. Despite this efforts, complex generic drug development support stakeholder confidence in the products’ interchangeability, bioequivalence requirements and clinical effectiveness [10].

In particular, physicians play a critical role in determining whether to prescribe originators or generics. Therefore, their preferences and perception on generics might be a critical factor that affects the degree to which generics are prescribed, clinical experience, education, and patient feedback [11]. Previous international studies have shown that while many physicians acknowledge the cost-effectiveness of generics, concerns remain about their therapeutic equivalence, especially in the treatment of severe or chronic conditions [12]. However, rising pharmaceutical costs for chronic / long term therapies continue to challenge both health systems and households, particularly in low- and middle-income countries. High out-of-pocket expenditure (OOPE) on medicines continues to be a major driver of health-related financial hardship accounting for nearly 40% of total health spending worldwide including Indian economy [13]. From an economic standpoint, the promotion of generic drugs represents a cost-effective strategy for reducing OOPE [14]. Globally, India is a major supplier of generic medications, providing 20% of the worldwide supply by volume and fulfilling around 60% of global vaccination demand [15].

Healthcare providers and patients generally have negative views of generic medical products. Complicated regulatory hurdles that make it difficult to enter the market, and murky pricing that makes people worry about whether or not they will be able to afford these lifesaving medications [16]. A cross-sectional survey study in India focused on doctors from tertiary-care teaching hospitals, showed that participants had poor knowledge of generic drugs. Many of them had misunderstandings about the similarity of active ingredients, therapeutic equivalence and quality consistency evaluation of generic medicines [17]. From Hadia et al., study it has seen that most of the physician were unaware about some regulatory requirements (like patent and exclusivity issues, preclinical and clinical study related aspects etc.) and 72% of doctors believe that generic product was manufactured in a poor quality to the brand-name medicines [18]. To tackle these intricate problems, agencies should encourage transparency and information sharing among healthcare providers which can be achieved through the establishment of public databases, continual medical education and stakeholder engagement forums that finally increase global healthcare access to lifesaving treatments [16]. Similarly several knowledge, attitude, and practice (KAP) studies has shown the knowledge gap among physician regarding generics [19, 20]. Complex generics represent a more advanced category of generic drugs. While they are designed to replicate the therapeutic effect of a brand-name counterpart, they involve additional scientific, manufacturing, or regulatory complexities [21]. However, there is limited information and studies regarding the KAP of doctors related to complex generic medications, especially in the Indian setting.

Assessing the viewpoints of physicians is therefore essential for designing educational initiatives and assisting in the development of adequate policy plans meant to increase the prescription of generic medications. Therefore, using a cross-sectional survey approach, the current study attempts to evaluate physicians’ knowledge of complex generic medications throughout India. It is anticipated that this study will assist policymakers, regulators, and academic institutions to develop evidence-based strategies targeted at fostering confidence, facilitating usage, and encouraging cautious intake of complex generic medications in routine clinical practice by identifying the gaps that currently exist and the important factors influencing the prescribing tendency.

## Methods

### Study Design and Procedure

A cross-sectional observational online anonymous survey study carried out over a period of 5 months from October 2025 through February 2026. Participants were recruited from most of the states of India (nearly 21 states) without providing any incentive for their participation. The voluntary survey was carried out among 100 physicians working in government or private hospitals, including those running their own clinics, across all over India. A snowball/ convenience sampling method was employed using a pretested, self-administered online questionnaire developed in Google forms, [22] as it was not feasible to reach physicians across India. The questionnaire was pilot-tested among 10 physicians before the survey link was sent to participants through social media platforms (WhatsApp groups, LinkedIn, and Instagram). The questionnaire required approximately 10 min to complete.

### Ethical Considerations

The Ethical Review Panel of the Kasturba Medical College and Kasturba Hospital Institutional Ethics Committee (Reg No: ECR/146/Inst/KA/2013/RR-19) and Clinical Trial Registry of India (CTRI) registration (Reference No: REF/2024/01/077272) approved the study. All methods performed on human participants have been conducted in accordance with the ethics guidelines set forth by the IEC, as well as in adherence to the Declaration of Helsinki (1964) and its subsequent amendments. Participation was voluntary, and anonymity was maintained throughout the process. Informed consent for voluntary participation was obtained, and no personal identifiers were recorded. All the responses were anonymized before analysis to guarantee objectivity and protect the data.

### Study Participants and Sample Size

Physicians from different states were invited to take part in this study. The sample size was determined by using web based sample size calculator wnarifin.github.io functions for a population survey [23]. To calculate an appropriate representative sample based on the standard deviation from pilot study, the Cochran formula was used with a precision of 0.1 at a 95% confidence level (α = 0.05) for a tool with an expected drop-out/non-response of 20% [24]. Accordingly, a target sample size of 100 physicians was estimated to demonstrate a précised exploratory survey result. Accordingly, the survey portal was closed when the number of responses exceeded the minimum required.

### Sampling Procedure

Participants were recruited using convenience and snowball sampling techniques due to the practical constraints of recruitment in the physician, whereby we value their service and time. Additionally although the study employed a non-probability sampling approach for exploratory evidence generation, efforts were made to minimize potential bias by collecting data from all parts of India. Furthermore, because these products are generic in nature, many Indian physicians has a belief that these products were only dispense in government hospital. Actually cost of complex generics are nearly similar to branded drugs and is also equally sold in private hospitals, to avoid bias the data were collected from both private and government hospital setting.

### Inclusion and exclusion criteria

The participants were selected only if they were registered with the relevant national council and those provide consent to participate. Licensed physician who are not practicing in clinical settings and those not currently working in India were excluded from this survey.

### Questionnaire Preparation, validation and design

The questionnaire for this study was developed after reviewing other studies on the same subject extensively [25–28] and after discussion among the co-authors. Questionnaire employed in the study is an original instrument created by the authors and is copyrighted (Certificate number LD-20260190067). A group of five physicians with a long experience in clinical practice and academia content validate the questionnaire, checked and commented on the credibility and appropriateness of survey questions. The Content Validity Index (CVI) was used to check for content validity. It was observed that the questionnaire had content validity as each item had good relevance score based on expert assessment (CVI score 0.92). In addition face validation was done among ten physician using a three-point Likert scale and modify the question according to the expert comments. After the modification, internal consistency reliability was assessed using Cronbach’s alpha coefficient, and the questionnaire exhibited good internal consistency (Cronbach’s alpha = 0.927). Then the third author transformed the survey into Google Forms which could be accessed only by the first and third authors. Google Forms was set to not include multiple submissions from the same individual. The English questionnaire was piloted and reviewed in a new group of ten physician, randomly chosen, to help understand and clarify questions, thus producing reliable results. Sequentially, the review generated minor changes; thus, the validation database was reset before the link was disseminated to participants.

The questionnaire consisted of 52 items designed as yes/no, open ended, Likert-scales and multiple-choice questions divided into two sections: demographic characteristics (1–11, including gender, specialization, type of organization, place of work, etc.), and KAP question (12–52). In the knowledge section was closed-ended and multiple answers were possible for some questions. The demographic section had eleven questions with multiple and short answer responses. Section 2 had eight questions which is testing the knowledge level of physician about complex generics. The attitude towards prescribing complex generics was assessed by twenty five Likert scale questions to measure the level of agreement and two questions among them are multiple choice and rest 23 were likert scale each with five options. The practice questions were eight with equal number of multiple choices and likert scale questions. Some of the questions involved in KAP were separated to know the regulatory and quality perspective. For this reason, the questions were also categorized into separate regulatory and quality domains, thus allowing for an investigation of physicians perceptions of regulation, quality, and treatment confidence pertaining to complex generics. Scores were assigned based on predetermined scoring guidelines, and domain score totals were determined for statistical purposes. The full questionnaire is available in the appendix (Additional file 1: Appendix A). KAP scores were computed by summing the correct answers for each section. Participants’ KAP levels were defined as ‘low’, ‘moderate’, and ‘high’ based on Bloom’s cutoff point [29].

### Data collection method

To collect data from physician, a web-based KAP cross-sectional online survey approach was used. The link to the Google survey was distributed individually via email, WhatsApp, and social media platforms (Instagram, Facebook, and LinkedIn). The survey’s aim, nature, and benefits were all listed on its opening page, which was followed by a consent page. The questionnaire page was accessible to those who clicked the agree button after giving their consent and agreement to participate in the study. To maintain the integrity of the data and prevent duplication of responses, a mechanism was implemented to allow only one response per participant. This ensures that each participant’s feedback is unique and contributes effectively to the study’s results. Participants were also encouraged to disseminate the survey to others using the snowball technique, thus reaching physician from all states of India. The response from the participants was entirely anonymous and voluntary.

### Data management, scoring and statistical data analysis of the collected data

Data were exported and cleaned in an excel worksheet where incomplete and duplicate responses were excluded from final datasheet. Missing data is minimal since the online survey require mandatory responses. Data were checked for completeness and consistency and the analysis was performed using Jamovi software. Both descriptive and inferential statistical analysis was employed in this study. Descriptive statistical analysis was used to summarize the demographic characteristics, and indices were created for knowledge, attitude, and practice by computing the correct answers of each section. Categorical data were reported as absolute numbers as well as proportions and described as frequency. Data that emerged from individual domains using the Likert scale were found non parametric (ordinal), whereas cumulative score of individual domains and total KAP score were considered as parametric (continous) because they are derived from summation of multiple likert scales. Since cumulative score is normal distribution, parametric test like independent t-test, one way ANOVA, Pearson correlation, and linear regression analysis were used for inferential analysis where the p-value of 0.05 or less was considered statistically significant in determining the outcome. Similar approach was used by Huh I and Gim J study verified that the Likert scale could be employed as a continuous variable in several parametric statistical methods [30]. Before performing any parametric statistics, normality of the domain scores was tested using the Shapiro-Wilk test. Composite scores for individual parameter were computed by taking the sum of all responses for each questionnaire item. While it is true that some composite scores had a statistically significant variation from normality when subjected to the Shapiro-Wilk test, the choice to run parametric analysis was made since the composite scores taken from several Likert scale questions are considered continuous variables, further pearson correlation and multiple regression analyses were done.

## Results

### Characteristics of physicians of the Study

A total of approximately 287 physicians from different parts of India were approached via email, professional networks and other online networking platforms. Among them 100 physicians participated in the survey voluntarily. The highest percentage of respondents (*n* = 29) was from North India (mainly from New Delhi, and Uttar Pradesh), whereas south (*n* = 26), east (*n* = 25), west (*n* = 20) Indian physicians were also equally contributed. Of the total sample, 56% were employee in government service hospitals and 44% were employee in private hospital/ have own clinic setup. A significantly higher mean practice score was found in junior physicians with below 5 years of experience compared to physician with higher than 10 years of experience. About 85% of the participant has not attended any training, CME, or workshop on complex generics, indicating limited professional education initiatives and training gap in this area (Table 1).

**Table 1:** Socio-demographic characteristics of physicians of the study (*n* = 100)

Demographic characteristics	Number
Gender	
Male	57
Female	43
Specialization	
General Practitioner (MBBS)	49
MD/MS/DM	40
Other	11
Total years of experience of practice	
1–5 years	78
5–10 years	14
11–20 years	5
More than 20 years	3
Experience abroad	
Yes	2
No	97
Type of organization	
Government	56
Private hospital/ Own clinic	44
Year of experience in current organization	
1–5 years	78
5–10 years	14
11–20 years	5
More than 20 years	3
Place of practice	
Urban	56
Rural	25
Semi-urban	19
Previous professional experience related to complex generic products	
Yes	37
No	63
Undergone any training, CME, or workshop on complex generics	
Yes	15
No	85
State currently practicing	
North India	29
West India	20
East India	25
South India	26

### Knowledge parameters

Despite of moderate awareness most of the participants showed limited knowledge of complex generics. Although 65% of the sample reported being aware about complex generics, only 16% reported to have an in-depth knowledge of those products and only 2% correctly identify all the example of complex generic products, highlighting a substantial gap in knowledge among practitioners (Table 2).

**Table 2:** Knowledge of physicians towards complex generics

Variable	*N* = 100
Are you aware of the concept of complex generics?	
No	19
Yes	16
Yes, I have aware but not in detail	65
Was the topic of complex generics covered in your medical academic course (UG/PG)?	
Maybe, not sure	30
No	51
Yes	19
What are complex generics according to your perspective?	
Generic medicine with multiple drugs	5
Generic version of drug device combination products	7
Generic medicines of advanced drug formulation	12
Generic medicines of orphan drug that need additional diagnostic tests before initiating treatment	2
Generic version of complex active ingredients, formulations, dosage forms, or routes of administration, or are complex drug-device combination products	58
Not sure	16
Do you believe complex generics differ from simple generics?	
No	4
Not sure	21
Yes	75
How do complex generics differ from simple generics as per your perspective?	
Complexities in formulation	25
Advanced delivery routes	21
For rare disease treatment	5
Multiple ingredients	13
Advanced generic product making difficulty in establishing clinical equivalence	21
Not sure	15
Which among the following, according to you, can be termed as complex generics? (Select as many answers as applicable)	
All wrong	13
One correct	72
Two correct	4
Three correct	4
Four correct	5
All correct	2
How often do you hear of Indian marketed complex generic products in your daily practice?	
Never	9
Rarely	42
Occasionally	35
Frequently	13
Very frequently	1
Do you agree whether complex generics need extra regulatory scrutiny?	
Strongly disagree	4
Disagree	4
Neutral	19
Agree	47
Strongly agree	26

### Attitude parameters

**Fig. 1:**
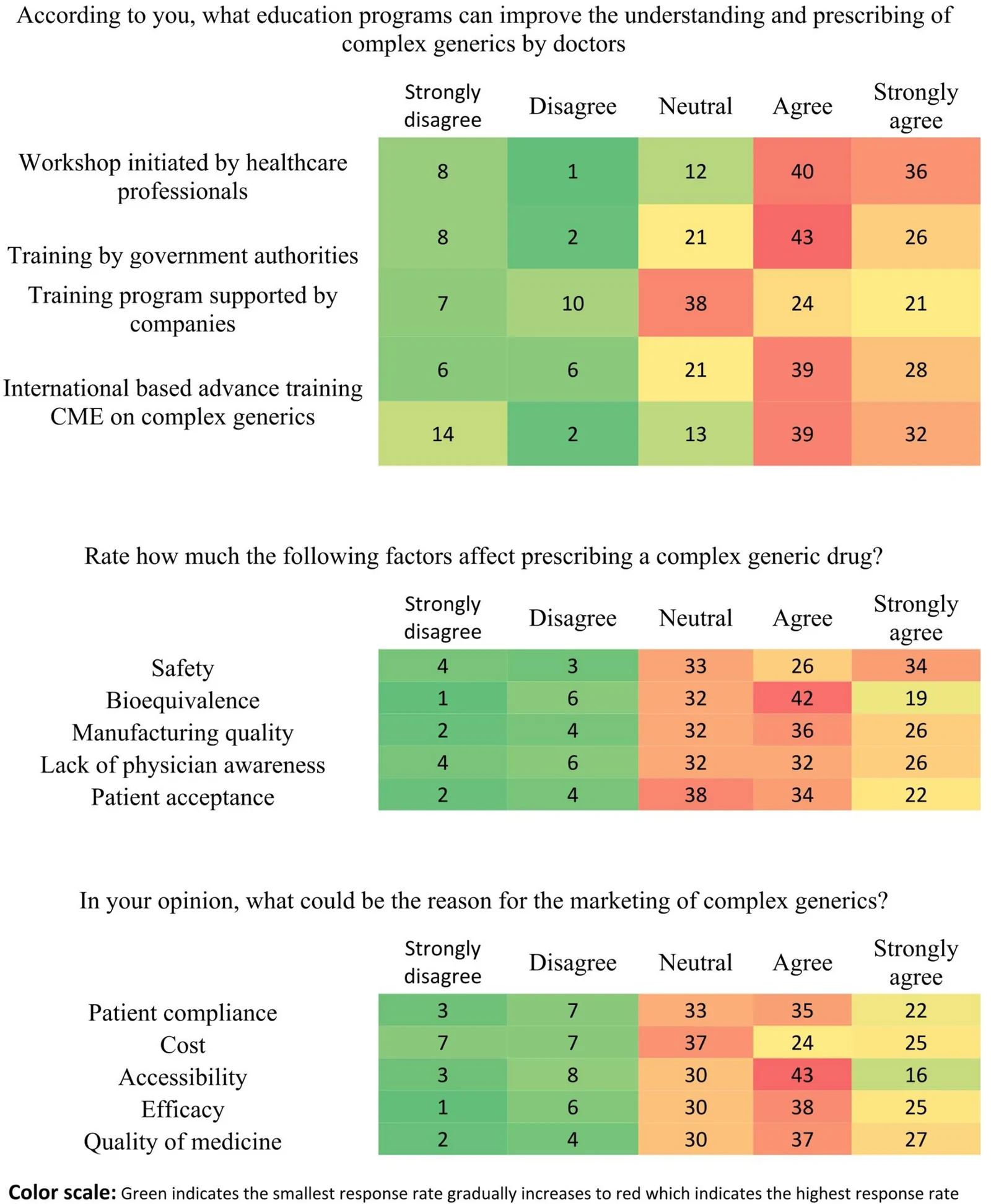
Heatmap showing responses related to the attitude of physician towards educational programs, prescribing behavior and acceptance

**Fig. 2:**
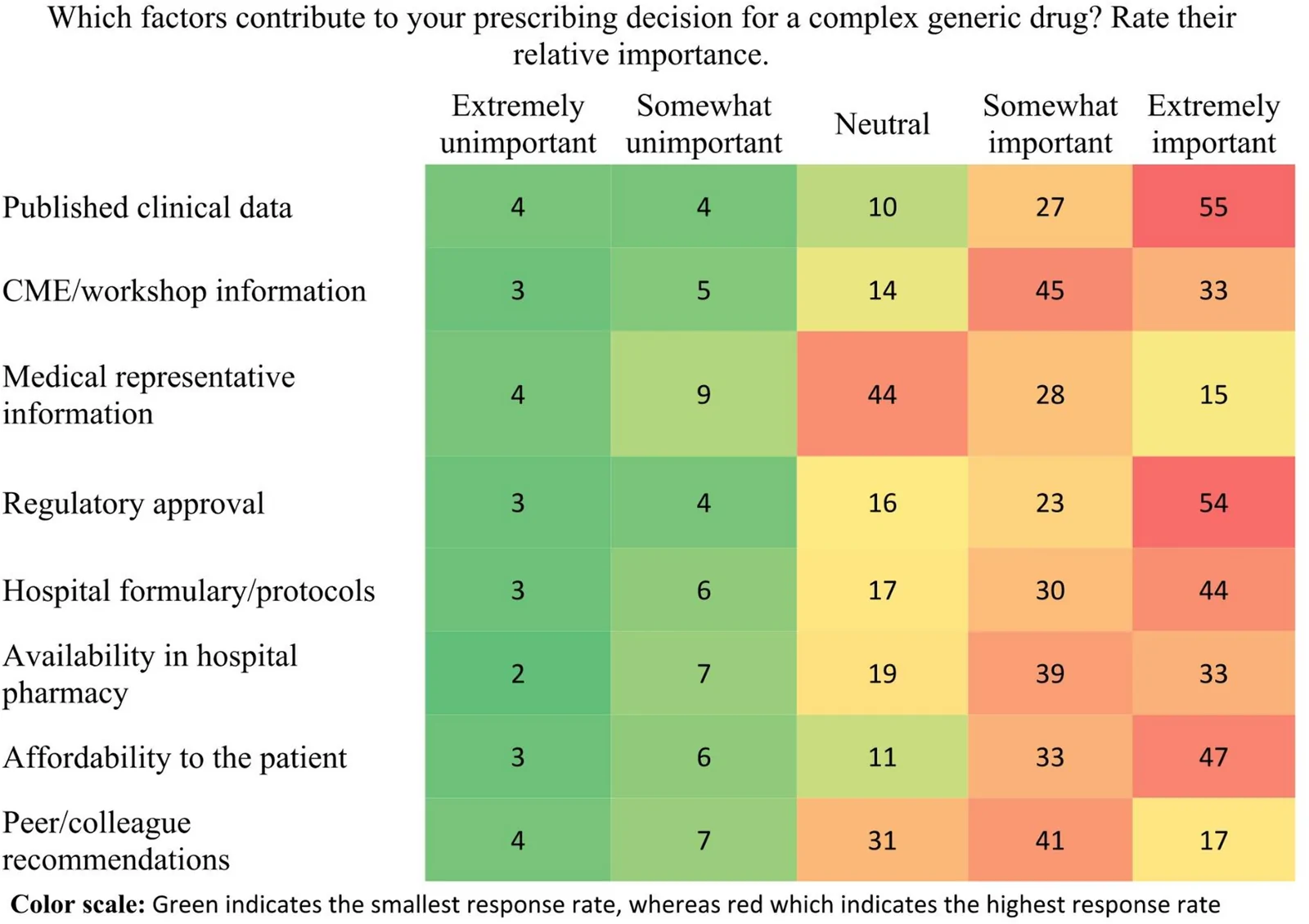
Heatmap showing responses related to the attitude of physician towards factors contributing prescribing decision making of complex generic drug

Positive attitudes towards educational interventions to improve the understanding of complex generics were reported (Figs. 1 and 2). Positive responses were obtained for workshops arranged by healthcare professionals (76%), governmental trainings (69%), international based advance training (67%), and CME training (71%) (Fig. 1). In terms of physician confidence regarding quality, safety, and efficacy of complex generics was modest. Nearly 49% of respondents stayed neutral, whereas only 26% reported confident in using complex generics (Table 3). Safety (60%), bioequivalence (61%), efficacy (63%), manufacturing quality (62%) and quality of medicine (64%) were identified as important criteria in decision-making process. Published clinical data (82%), CME/workshop information (78%), regulatory approval of generics (77%), affordability (80%), hospital formularies or protocol (74%) and availability in hospital pharmacy (72%) were deemed as essential criteria in prescribing decisions (Fig. 2).

**Table 3:** Other questions related to attitude of physicians towards complex generics

Variable	Sub-variable (code)	Number (*n*)= Percentage (%) Since sample size = 100
How confident are you about the quality, safety, and efficacy of complex generics?	Extremely not confident	1
	Not so confident	24
	Neutral	49
	Somewhat confident	24
	Extremely confident	2
In your opinion, what could be the best value assessment technique for complex generics?	Cost-utility analysis	7
	Cost-consequence analysis	10
	Cost-benefit analysis	41
	Cost-minimization analysis	7
	Cost-effective analysis	35

### Practice parameters

Prescribing complex generics are very limited in India, around 53% of the participants prescribed complex generics to less than five patients per month. Research articles from scientific journals emerged as the highest source of information to assure safety and quality of complex generics (42%), followed by information from colleagues/ peers/Key opinion leaders (23%) and conferences/workshops/seminars (18%). Almost 62% of the participants were involved either not at all or slightly in complex generics products during their current practice. Cost appeared as a crucial factor in prescribing complex generics by about 79% of the participants. Similarly, clinical study reports (80%) and patient compliance (81%) emerged as the extremely or very important factors during prescribing complex generics as depicted in Table 4.

**Table 4:** Practice parameters of physicians towards complex generics

Variable	Sub-variable (code)	Number (*n*)= Percentage (%) Since sample size = 100
Appropriately in how many patients in a month do you prescribe complex drugs such as liposomal injection such as Doxin- Doxorubicin, Dry powder inhaler Symbicort- Budesonide+Formoterol, Restasis- Generic cyclosporin emulsion, Copaxone- Generic glatiramer acetate?	Less than 5 patients	53
	5–10 patients	22
	10–20 patients	15
	20–30 patients	1
	More than 30 patients	9
What source of information do you rely on to assure safety and quality of complex generics while prescribing?	Detailing from medical representative	5
	Research articles published in scientific journal	42
	Conferences, workshops and seminars	18
	Colleagues/peers/Key opinion leaders	23
	Continuing Medical Education	12
To what level does your current job involve practical or hands-on experience with complex generic products?	Not at all involved	20
	Slightly involved	42
	Moderately involved	30
	Highly involved	5
	Fully involved	3
How important is the cost of complex generics when prescribing them to a patient?	Extremely not important	2
	Somewhat not important	3
	Neutral	16
	Somewhat important	32
	Extremely important	47
What factors are important to you while prescribing complex generics? [Cost]	Extremely not important	2
	Somewhat not important	5
	Neutral	12
	Somewhat important	37
	Extremely important	44
What factors are important to you while prescribing complex generics? [Clinical study report]	Extremely not important	2
	Somewhat not important	4
	Neutral	14
	Somewhat important	19
	Extremely important	61
What factors are important to you while prescribing complex generics? [Patient compliance]	Extremely not important	4
	Somewhat not important	1
	Neutral	14
	Somewhat important	27
	Extremely important	54
What factors are important to you while prescribing complex generics? [Regularity of medical representative visit]	Extremely not important	6
	Somewhat not important	11
	Neutral	37
	Somewhat important	25
	Extremely important	21

### Regulatory parameters

Most participants were knowledgeable concerning the regulatory significance of complex generics. 77% of the respondents believed that regulatory approval was either somewhat or extremely important factor that contribute decision-making during prescribing. Moreover, 73% of the participants agreed or strongly agreed that complex generics needs extra regulatory scrutiny before approval, reflecting regulatory gap in product complexity and therapeutic equivalence (Table 5).

**Table 5:** Regulatory and quality parameters of physicians towards complex generics

Regulatory Variable	Sub-variable (code)	Number (*n*)= Percentage (%) Since sample size = 100
What are complex generics according to your perspective?	Generic medicine with multiple drugs	5
	Generic version of drug device combination products	7
	Generic medicines of advanced drug formulation	12
	Generic medicines of orphan drug that need additional diagnostic tests before initiating treatment	2
	Generic version of complex active ingredients, formulations, dosage forms, or routes of administration, or are complex drug-device combination products	58
	Not sure	16
Which among the following, according to you, can be termed as complex generics? (Select as many answers as applicable)	All wrong	13
	One correct	72
	Two correct	4
	Three correct	4
	Four correct	5
	All correct	2
Which factors contribute to your prescribing decision for a complex generic drug? Rate their relative importance. [Regulatory approval]	Extremely unimportant	3
	Somewhat unimportant	4
	Neutral	16
	Somewhat important	23
	Extremely important	54
Do you agree whether complex generics need extra regulatory scrutiny?	Strongly disagree	4
	Disagree	4
	Neutral	19
	Agree	47
	Strongly agree	26

Quality Variable	Sub-variable (code)	Number (*n*)= Percentage (%) Since sample size = 100
How confident are you about the quality, safety, and efficacy of complex generics?	Extremely not confident	1
	Not so confident	24
	Neutral	49
	Somewhat confident	24
	Extremely confident	2
What source of information do you rely on to assure safety and quality of complex generics while prescribing?	Detailing from medical representative	5
	Research articles published in scientific journal	42
	Conferences, workshops and seminars	18
	Colleagues/peers/Key opinion leaders	23
	Continuing Medical Education	12
Rate how much the following factors affect prescribing a complex generic drug? [Safety]	Strongly disagree	4
	Disagree	3
	Neutral	33
	Agree	26
	Strongly agree	34
Rate how much the following factors affect prescribing a complex generic drug? [Bioequivalence]	Strongly disagree	1
	Disagree	6
	Neutral	32
	Agree	42
	Strongly agree	19
Rate how much the following factors affect prescribing a complex generic drug? [Manufacturing quality]	Strongly disagree	2
	Disagree	4
	Neutral	32
	Agree	36
	Strongly agree	26
In your opinion, what could be the reason for the marketing of complex generics? [Efficacy]	Strongly disagree	1
	Disagree	6
	Neutral	30
	Agree	38
	Strongly agree	25
In your opinion, what could be the reason for the marketing of complex generics? [Quality of medicine]	Strongly disagree	2
	Disagree	4
	Neutral	30
	Agree	37
	Strongly agree	27

### Quality parameters

With respect to confidence in the safety, efficacy, and quality of complex generics, 49% of the respondents were neutral, while only 26% of them felt somewhat or extremely confident. Safety, bioequivalence, and manufacturing quality were the three most crucial factors affecting their prescription decisions among 60%, 61%, and 62% of respondents respectively. Research papers published in scientific journals emerged as the main source consulted by 42% of respondents for ensuring safety and quality, followed by colleagues/peers/ key opinion leaders (23%), and conferences or workshops (18%). On the other hand, 5% preferred medical representatives as sources of these facts. On top of that, 63% of respondents found efficacy as important for the marketing of complex generics while 64% thought that the quality of medicine is also a crucial determinant (Table 5).

### Bivariate analysis of Domain Scores

The bivariate analysis indicated statistically significant correlations between some demographic variables and the KAP scores as given in Table 6. Those with prior experience in the area of complex generics recorded significantly higher scores in knowledge (*p* = 0.025), attitude (*p* = 0.036), practice (*p* = 0.013), quality (*p* = 0.007), and regulatory (*p* = 0.009). Likewise, those who had previously attended any form of training or CME or workshop on complex generics were found to have significantly higher knowledge scores (*p* = 0.011). The years of experience in the current place of employment correlated significantly with practice (*p* = 0.038) and quality (*p* = 0.017). On the other hand, place of practice was found to correlate significantly with regulatory perception scores (*p* = 0.032). Experience will facilitate familiarity with the product and regulation thereof. Thus, training is essential for addressing the identified gaps in knowledge and practice. Practice scores were significantly related to gender (*p* = 0.046) since male respondents exhibited comparatively high practice scores than female respondents. This indicates that there may be some difference in clinical exposure, patient care, medical specialties, or involvement in the prescription of complex generics. However, since no gender differences existed with regard to knowledge, attitude, quality, or regulation, gender may not be a determining factor for complex generic perception.

**Table 6:** Bivariate analysis of factors associated with knowledge, attitude, practice, quality and regulatory parameters domain scores towards complex generics

Category	K (Mean ± SD)	A (Mean ± SD)	*P* (Mean ± SD)	Q(Mean ± SD)	*R*(Mean ± SD)
Gender	0.326^*^	0.340^*^	**0.046** ^*^	0.264^*^	0.687^*^
Female	19.1 ± 2.56	92.0 ± 13.6	26.6 ± 3.74	24.4 ± 3.85	15.0 ± 1.83
Male	18.5 ± 3.55	95.4 ± 20.2	28.2 ± 4.30	25.4 ± 4.87	14.8 ± 2.92
Specialization	0.897^*^	0.292^*^	0.691^*^	0.805^*^	0.679^*^
General Practitioner (MBBS)	18.7 ± 3.54	91.6 ± 19.4	27.5 ± 4.07	24.7 ± 4.80	14.7 ± 2.73
MD/MS/DM	18.9 ± 2.83	94.9 ± 15.4	27.3 ± 4.55	25.0 ± 3.77	15.0 ± 2.42
Other	19.1 ± 2.70	100.7 ± 16.6	28.3 ± 2.87	25.9 ± 5.54	15.2 ± 1.72
Total years of experience of practice	0.473^*^	0.743^*^	0.762^*^	0.182^*^	0.348^*^
1–5 years	18.7 ± 3.07	93.4 ± 17.4	27.4 ± 4.09	24.6 ± 4.33	15.0 ± 2.44
11–20 years	20.8 ± 3.19	100.4 ± 16.0	29.2 ± 5.22	28.6 ± 3.21	15.6 ± 1.14
5–10 years	18.6 ± 3.71	96.8 ± 16.8	28.2 ± 3.45	26.1 ± 4.92	14.2 ± 2.72
More than 20 years	17.0 ± 2.65	84.0 ± 33.4	25.3 ± 7.23	23.3 ± 6.11	12.0 ± 3.61
Experience abroad (if any)	0.566^*^	0.867^*^	0.171^*^	0.418^*^	0.717^*^
No	18.8 ± 3.18	93.9 ± 17.8	27.4 ± 4.12	24.9 ± 4.47	14.8 ± 2.53
Yes	17.5 ± 2.12	95.5 ± 10.6	31.5 ± 3.54	27.5 ± 4.95	15.5 ± 0.707
Type of organization	0.347^*^	0.125^*^	0.874^*^	0.204^*^	0.623^*^
Government	19.0 ± 3.45	91.6 ± 19.8	27.6 ± 4.28	24.5 ± 4.62	15.0 ± 2.80
Private hospital/Own clinic	18.4 ± 2.71	97.1 ± 13.8	27.5 ± 3.97	25.6 ± 4.21	14.7 ± 2.04
Year of experience in current organization	0.404^*^	0.400^*^	**0.038** ^*^	**0.017** ^*^	0.446^*^
1–5 years	18.8 ± 3.18	93.3 ± 17.4	27.2 ± 4.059	24.7 ± 4.33	14.9 ± 2.46
11–20 years	22.3 ± 3.06	108.3 ± 11.0	32.7 ± 2.309	30.0 ± 1.00	16.0 ± 1.00
5–10 years	17.8 ± 2.70	93.3 ± 21.8	28.0 ± 4.714	25.4 ± 5.62	14.3 ± 3.30
More than 20 years	18.0 ± 2.83	102.0 ± 17.0	29.5 ± 0.707	26.0 ± 5.66	14.0 ± 1.41
Place of practice	0.266^*^	0.181^*^	0.108^*^	0.106^*^	**0.032** ^*^
Rural	18.1 ± 3.46	87.1 ± 23.2	26.0 ± 4.62	23.2 ± 4.94	13.6 ± 2.75
Semi-urban	18.3 ± 3.07	97.5 ± 14.1	27.4 ± 3.39	25.9 ± 4.01	14.9 ± 2.44
Urban	19.3 ± 3.03	95.8 ± 15.3	28.3 ± 4.00	25.4 ± 4.26	15.4 ± 2.25
Which state do you currently practice in?	0.796^*^	0.129^*^	0.422^*^	0.519^*^	0.197^*^
East India	19 ± 3.79	88.3 ± 22.0	26.4 ± 4.15	24.0 ± 5.20	14.0 ± 2.56
North India	18.4 ± 3.29	99.3 ± 14.4	28.4 ± 4.34	25.7 ± 4.40	15.4 ± 2.03
South India	18.7 ± 2.48	96.2 ± 12.7	27.5 ± 3.43	25.4 ± 3.48	14.8 ± 2.36
West India	19.3 ± 3.06	90.3 ± 19.9	27.6 ± 4.60	24.4 ± 4.78	15.1 ± 3.09
Have you ever had any previous professional experience related to complex generic products?	**0.025** ^*^	**0.036** ^*^	**0.013** ^*^	**0.007** ^*^	**0.009** ^*^
No	18.2 ± 2.72	91.1 ± 17.5	26.7 ± 3.95	24.0 ± 4.38	14.4 ± 2.37
Yes	19.7 ± 3.65	98.8 ± 17.2	28.9 ± 4.15	26.5 ± 4.23	15.7 ± 2.53
Have you ever undergone any training, CME, or workshop on complex generics?	**0.011** ^*^	0.412^*^	0.279^*^	0.218^*^	0.747^*^
No	18.4 ± 2.84	93.3 ± 17.4	27.3 ± 4.16	24.7 ± 4.40	14.9 ± 2.44
Yes	20.7 ± 4.22	97.4 ± 19.2	28.60 ± 3.91	26.3 ± 4.79	14.7 ± 2.89

*Indicates p-value and bold *p* value indicates significance of sociodemographic parameters that affect KAP towards complex generics prescription

Gender, Experience abroad, type of organization, previous professional experience, and training: via Student t test

Specialization, total years of experience, experience in current organization, place of practice, state currently practice: via ANOVA

### Correlation study

**Fig. 3:**
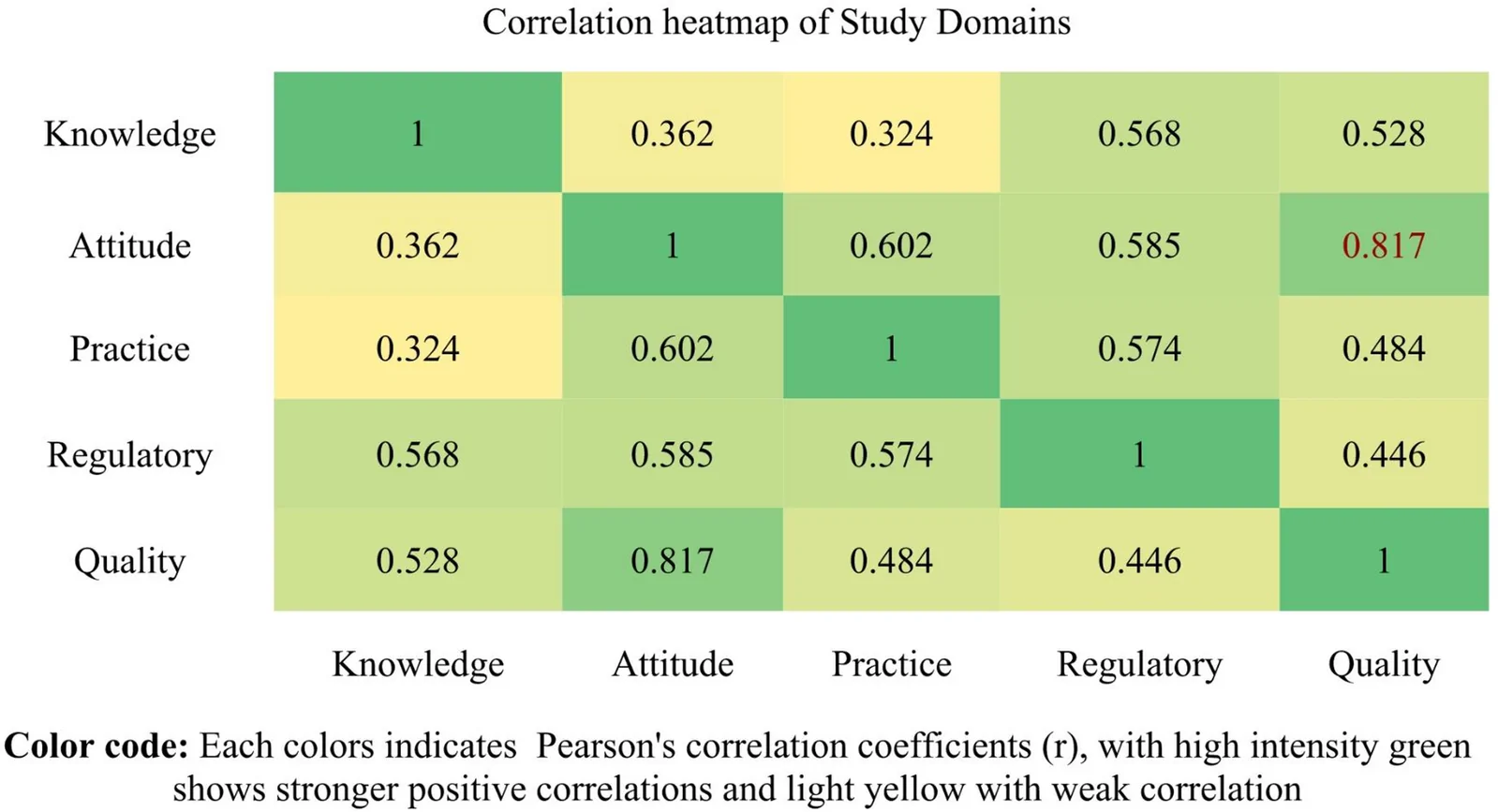
Pearson’s correlation matrix of knowledge, attitude, practice and regulatory correlation scores

Positive correlations were identified among all the variables considered in the current study with correlation analysis (Fig. 3). Knowledge correlated moderate-positively with attitude (*r* = 0.362, *p* < 0.001), practice (*r* = 0.324, *p* < 0.001), regulatory perception (*r* = 0.568, *p* < 0.001), and quality perception (*r* = 0.528, *p* < 0.001). Attitude correlated positively with quality perception (*r* = 0.817, *p* < 0.001), moderate-positively with practice (*r* = 0.602, *p* < 0.001), and regulatory perception (*r* = 0.585, *p* < 0.001). Practice exhibited a moderately high positive relationship with regulatory perception (*r* = 0.574, *p* < 0.001) and quality perception (*r* = 0.484, *p* < 0.001). Furthermore, regulatory perception indicated a moderately positive relationship with quality perception (*r* = 0.446, *p* < 0.001).

### Results of Regression analysis

#### Regression analysis of factors associated with total score of KAP

The regression results of the adjusted regression model indicated that doctors with more than 20 years of work experience exhibited lower total KAP scores when compared with their colleagues who have been working for only 1 to 5 years (β = -70.11, *p* = 0.003). Doctors with more than 20 years of work experience at the present organization exhibited higher KAP scores (β = 86.68, *p* = 0.002). Urban doctors exhibited higher KAP scores compared with rural doctors (β = 11.02, *p* = 0.05). North Indian doctors shown higher KAP scores compared with other parts of India (β = 12.91, *p* = 0.05). Prior professional experience in complex generics increased KAP scores (β = 10.76, *p* = 0.03). Training/CME/workshop on complex generics has a positive impact on KAP score when analyzing using unadjusted regression model (β = 11.2, *p* = 0.012). Linear regression results of both adjusted and unadjusted regression model were given in Table 7.

**Table 7:** Association of knowledge, attitude, and practice scores with socio-demographic characteristics

Variable	Categories	Estimate	SE	t	*p*	Estimate	SE	t	*p*
		Unadjusted				Adjusted			
Intercept*						118.16	6.36	18.58	< 0.001
Gender	Male-Female	4.46	4.41	1.01	0.31	3.91	4.59	0.85	0.40
Specialization	MD/MS/DM -General Practitioner (MBBS)	3.23	4.65	0.68	0.49	1.44	5.01	0.29	0.77
	Other-General Practitioner (MBBS)	10.27	7.28	1.41	0.16	2.38	7.67	0.46	0.65
Total years of experience of practice	11–20 years-1- 5 years	10.89	10.08	1.08	0.28	-13.65	15.25	-0.89	0.37
	5–10 years-1- 5 years	4.13	6.34	0.63	0.52	3.51	7.67	0.31	0.76
	More than 20 years-1- 5 years	-13.18	12.86	-1.02	0.31	-70.11	23	-3.05	0.01
Experience abroad (if any)	Yes -No	4.35	15.66	0.28	0.78	-24.23	17.04	-1.42	0.16
Type of organization	Private hospital/Own clinic -Government	4.76	4.42	1.08	0.28	-1.66	4.78	-0.35	0.73
Year of experience in current organization	11–20 years − 1–5 years	23.99	12.76	1.88	0.06	37.83	21.01	1.8	0.08
	5–10 years-1- 5 years	-0.241	7.26	-1.13	0.97	4.83	8.88	0.54	0.59
	More than 20 years-1- 5 years	10.16	15.54	0.65	0.51	86.68	27.44	3.16	0.01
Place of practice	Semi-urban -Rural	12	6.51	1.84	0.07	8.9	6.64	1.34	0.18
	Urban-Rural	12.1	5.15	2.34	0.02	11.02	5.53	1.99	0.05
Which state do you currently practice in?	North India -East India	12.31	5.9	2.09	0.04	12.91	6.5	1.98	0.05
	South India-East India	8.55	6.05	1.41	0.16	11.58	6.28	1.90	0.06
	West India-East India	3.44	6.48	0.53	0.60	4.81	6.49	0.74	0.46
Previous professional experience related to complex generic products	Yes -No	11.2	4.4	2.56	0.01	10.76	4.88	2.20	0.03
Training/CME/workshop on complex generics	Yes -No	11.2	4.4	2.56	0.01	-2.78	6.41	-0.43	0.67

### Regression analysis of factors associated with total score of QR

Using adjusted regression analysis, it was found that respondents with prior professional experience associated with complex generics had significantly higher scores on QR (β = 3.87, *p* = 0.007). There were significantly high scores on QR for urban practitioners than for their rural counterparts (β = 3.53, *p* = 0.029). Those respondents with practice experience exceeding 20 years had significantly low scores on QR (β = -17.39, *p* = 0.01), while those with organizational experience exceeding 20 years had significantly high scores on QR (β = 19.83, *p* = 0.014). Linear regression results of quality and regulatory scores of both adjusted and unadjusted regression model were given in Table 8.

**Table 8:** Association of quality and regulatory scores with socio-demographic characteristics

Variable	Categories	Estimate	SE	t	*p*	Estimate	SE	t	*p*
		Unadjusted				Adjusted			
Intercept*						33.58	1.82	18.44	< 0.001
Gender	Male-Female	0.81	1.24	0.65	0.52	0.62	1.31	0.47	0.64
Specialization	MD/MS/DM -General Practitioner (MBBS)	0.63	1.39	0.48	0.63	-0.09	1.43	-0.06	0.95
	Other-General Practitioner (MBBS)	1.72	2.06	0.83	0.40	-0.32	2.22	-0.14	0.88
Total years of experience of practice	11–20 years-1- 5 years	4.58	2.81	1.63	0.11	1.42	4.37	0.32	0.75
	5–10 years-1- 5 years	0.67	1.77	0.38	0.71	0.81	2.20	0.37	0.71
	More than 20 years-1- 5 years	-4.28	3.59	-1.19	0.24	-17.40	6.59	-2.64	0.01
Experience abroad (if any)	Yes -No	3.26	4.40	0.74	0.46	-4.14	4.88	-0.85	0.399
Type of organization	Private hospital/Own clinic -Government	0.90	1.25	0.72	0.47	-0.54	1.37	-0.39	0.69
Year of experience in current organization	11–20 years − 1–5 years	6.4	3.60	1.77	0.08	4.24	6.02	0.70	0.48
	5–10 years-1- 5 years	0.1	2.05	0.05	0.96	1.88	2.54	0.74	0.46
	More than 20 years-1- 5 years	0.4	4.39	0.09	0.93	19.83	7.86	2.52	0.01
Place of practice	Semi-urban -Rural	4.05	1.81	2.24	0.03	3.21	1.9	1.69	0.09
	Urban-Rural	3.93	1.43	2.74	0.01	3.53	1.58	2.23	0.03
Which state do you currently practice in?	North India -East India	3.13	1.67	1.87	0.06	3.01	1.86	1.62	0.11
	South India-East India	2.23	1.71	1.30	0.20	3.06	1.74	1.75	0.08
	West India-East India	1.41	1.84	0.77	0.44	2.02	1.86	1.09	0.28
Previous professional experience related to complex generic products	Yes -No	3.82	1.22	3.13	0.01	3.87	1.4	2.77	0.01
Training/CME/workshop on complex generics	Yes -No	1.32	1.72	0.77	0.44	-1.41	1.84	-0.77	0.44

## Discussion

To best of our knowledge, the current study is among the first study from India to assess the knowledge, attitude and prescribing practices of physicians towards complex generic drugs from a pharmaceutical regulatory perspective. The study highlights a positive attitude towards accessible cost effective complex generic drugs even with a moderate knowledge and practical experience among physicians in India. Concerns relating to the quality, efficacy, equivalency, clinical reliability and others regulatory aspects were raised by most of the participants. This study further shows the significant influence of prescribing behavior towards the knowledge and attitude of physician, highlighting the importance of regulatory awareness even in clinical decision making.

The knowledge study revealed although most of the physician has adequate knowledge about the concept generic medicine, there is a gap exists in case of complex generics. Even basic knowledge exist, detailed understanding about the concept bioequivalence, substitutability, therapeutic equivalence and regulatory requirements for approval was limited in terms of complex generics. The similar outcomes were observed in previous studies conducted to access the perspection of physician towards simple generics. Studies conducted by Priyadarsini et al. [31], Hadia et al. [18] and Charan et al. [32] similarly reported that knowledge regarding efficacy, safety, bioequivalence and therapeutic failure of generics are low among physicians. The moderate score observed in this current study also highlights the limited experience or exposure to regulatory aspects of drug products. Since complex generics are advanced products requiring complex formulation and bioequivalence studies, the lack of knowledge could potentially impact prescription decisions.

Considering the deficiencies in knowledge highlighted, there is a need for the inclusion of information on issues concerning complex generic medications in both undergraduate and graduate medical courses. Considering the deficiencies in knowledge highlighted, there is a need for the inclusion of information on issues concerning complex generic medications in both undergraduate and graduate medical courses. Efforts need to be taken from their academic career as well throughout the professional life via continual medical education or training activities related to the complex generic products as well as their regulations that will significantly impact knowledge about optimal use of medicine and improve health-care budgets.

Despite of gap in knowledge, participants had a positive attitude towards prescribing complex generics because of public health benefits. Similar findings are seen in previous studies conducted by Lewandowski et al. [33] and Nakipov et al. [34], where despites of the concerns about the effectiveness and safety of generics drugs physicians strongly recommends generics due to the reduction of healthcare cost. Complex generics have active ingredients, formulations, methods of administration, or routes of administration which are far more complicated than the usual generics, making pharmaceutical and therapeutic equivalence harder to attain. Consequently in the current study, physicians raise concerns regarding safety, efficacy, manufacturing quality, interchangability and therapeutic equivalency. Neutral opinion of physicians in terms of confidence in complex generics QSE is likely related to the insufficient amount of data available and lack of explanation concerning regulatory procedures and assessment. Persistent uncertainty may completely affects confidence, trust and willingness to prescribe the product. Hospital formulary committees have a key role to play in that they can rigorously analyze the literature on complex generics and make institutional recommendations on how to properly use these medications. This can lead to greater confidence on the part of prescribers.

From the practice assessment it seems that the prescription of complex generics by physicians was still quite limited. Low prescribing rate might be because of less penetration of the product in the market, less familiarity or lack of organizational support with respect to the complex generics. The study also demonstrate the prescribing decision not solely depend on the economic benefit, but also based on different influencing factors like clinical reliability and patient compliance. Similar observation has been reported in Sarnola et al. [35] study on biosimilars where doubts related to safety, efficacy and immunogenicity on biosimilars has reduce the acceptance and prescribing manner even with a high attitude score. Less familiarity about the data produced to agency for market approval may therefore contribute to caution adoption pattern. Physicians largely relied on scientific literature and information from credible sources before prescribing such products. This indicates the relevance of evidence-based medicine in decision-making by physicians.

A positive correlation between knowledge, attitude and practice scores implies that doctors who use complex generics in their practice may build greater confidence about the efficacy, safety, and manufacturing quality of such medicines due to hands-on experience. Similarly positive attitude towards cost and accessibility of complex generics were improved by physician acceptance of the product. In addition, the connection between practice and regulatory perception shows that doctors with extensive practice can build greater awareness about the regulation of complex generics.

From the regression analysis, identified significant knowledge and attitude predictors that improve the physician acceptance and prescribing. Such findings uphold the theoretical framework of KAP research, whereby increased knowledge has a positive impact on perceptions, which then impacts professional conduct. Additionally, the multiple regression analysis emphasizes the importance of training, workshop, research projects, policy discussions, and CMEs to build trust and confidence towards such products. Strengthening the opportunities to stay updated with scientific and regulatory assessment for quality assurance and market approval may improve the prescribing practices.

This study further possesses important health implication to reduce healthcare burden. Complex generics are improving the access to cost effective option to high cost branded medicines. Increase usage of complex generics may reduce healthcare financial burden of patients with improved quality and therapeutic outcome especially countries like India where healthcare expenditure is high. Despite of economic benefits, insufficient knowledge, awareness regarding the product, clinical outcome, and regulatory requirements hinders the prescribing attitude of physician. Increasing physician knowledge and encouraging rational prescription practices could, therefore, help to achieve broader public health goals concerning the affordability of care, equitable drug access, and sustainable healthcare systems.

From regulatory perspective, the current study emphasized the importance of strengthening the pharmaceutical regulatory based training to physicians. Regulatory transparency in terms of bioequivalence evidence, market approval data requirements, quality and safety assessment procedures, and post marketing surveillance enhance the physician confidence towards these classes of generic products. The findings from the study highlight the strong gap in communication between the regulatory agencies and pharmaceutical industries with physician. Further reinforcement of physicians trust can be achieved through enhanced communication about the scientific evidence behind the approval of complex generics. Educational tools, scientific webinars, frequently asked questions, and publicly available regulatory assessments could contribute to such an understanding of the scientific evidence underlying the drug approval process. The development of physician based regulatory model need to be initiated by regulators or industrial stakeholders will enhance the physician confidence and further support clinical practices to reduce healthcare financial burden. Prescribing policies from national and organizational sources should provide more specific guidelines on the use and interchangeability of complex generic drugs. This might decrease the level of ambiguity for the physician and help standardize clinical practices.

In summary, the results of this exploratory study reveal critical deficiencies in the education and regulation of physicians regarding complex generic drugs. Bridging such deficiencies by way of better medical education, more CME activities, prescription guidelines, more involvement of formulary committees in hospitals, and better communication efforts on the part of regulators can help promote informed decision-making by physicians and the adoption of complex generic drugs. Future multicenter research utilizing probability samples is indicated in order to confirm the results and intervention efficacy.

### Limitation and strength

There are a few limitations to this study. The use of convenient sampling as well as snowball sampling on the online medium can cause selection bias among other problems, which will affect the sample representativeness. Although the present study covered participants from 21 states of India, the limited sample size due to time constraints as well as the use of non-probability sampling method was considered as a possible weakness (*n* = 100) which affect the generalizability of the research outcome. Hence, the results should be interpreted to be exploratory and not representative of the nation as a whole. Besides, this study was dependent on self-reported answers that could suffer from either recall bias or social desirability bias. Some subgroup analyses have used a low number of participants, especially the senior physicians who have worked for more than 20 years; thus, these results including multiple regression estimates should be interpreted carefully. Additionally, the survey was an anonymous online google form based study which restricts the direct access to the participants who are with limited digital access. Additionally, senior experienced participant counts was lower, may be because older participants may less engaged with online platforms or because of their busy work schedules. Juniors physicians were larger in proportion which may influence the study reports. Inter-domain correlations played a role in the partial overlap in the KAP, regulation, and quality parameters because some questions pertained to more than one construct domain. Overall, since it is a cross-sectional design, causality between physician attributes and KAP scores could not be proven. Based on these limitations, the future studies can be done on larger number of samples with inclusion of balanced number of senior physicians. Multi centric study using mixed mode both online and offline data collection method may give deeper insights.

Additionally, the current study has several strengths since it is among the first study to assess the impact of KAP of physician in prescribing behavior towards the complex generic drug products from clinical regulatory perspective. Professionals from various geographic regions of India and diverse specialization resulted in greater regional representation and more diverse perspectives on complex generics. Furthermore the correlation and regression study gives deeper insights into the factors influencing physician prescribing behavior.

## Conclusion

The current exploratory multicentric study across multiple Indian states concludes that physicians possess moderate knowledge about complex generics due to limited scientific and regulatory awareness about the product. Most participants shows positive attitude towards the need for additional regulatory scrutiny and awareness training program initiatives to develop prescribing confidence regarding the equivalency, SQE, and clinical reliability. Prior experience, training/ workshop, and professional exposure with complex generics were significantly associated with improved KAP score as well as QR score. Furthermore, correlation analysis concludes that improved knowledge was associated with positive attitude, prescribing practices, and enhancing confidence in regulatory and quality aspects. The gap between the awareness and practical familiarity with marketed product highlight the importance of communication initiative between the regulatory, industries and physician to improve the understanding of the product, confidence on QSE and acceptance in clinical practice. Future studies using probability based sampling needs to be conducted to confirm the current study findings and give a national representiveness. In addition, the findings of the present study emphasize the need for educational initiatives from the academic level, CME programs, regulatory awareness workshop to increase evidence based decision making.

## Supplementary Information

Supplementary Material 1.

Supplementary Material 2.

## Data Availability

The datasets used in the study have been kept confidential as these are being used for a PhD research project which is currently ongoing. The data cannot be shared because of the privacy of the research. However, the data can be accessed by contacting the corresponding author for this purpose after the completion of the course.

## Abbreviations

KAP: Knowledge, attitude and practice
US FDA: US Food and Drug Administration
pre-ANDA: pre- Abbreviated New Drug Application
OOPE: Out-of-pocket expenditure
CTRI: Clinical Trial Registry of India
CVI: Content Validity Index
CME: Continual medical education
QSE: Quality, safety, efficacy
