# Supplementary material for: Knowledge, Attitudes and Practices regarding complex generic drugs among physicians in India: A cross-sectional survey
Source: BMC Public Health. 2026 Jul 30;26:3084. doi: 10.1186/s12889-026-28768-1 (PMC13711814; doi:10.1186/s12889-026-28768-1)
Supplement: Supplementary file 1 [file 12889_2026_28768_MOESM2_ESM.pdf]

Survey on knowledge, attitude and practice of physicians towards complex generics in India

* Indicates required question

KAP questions

1. Are you aware of the concept of complex generics? *

Mark only one oval.

- Yes
- Yes, I have aware but not in detail
- No

2. Was the topic of complex generics covered in your medical academic course (UG/PG)? *

Mark only one oval.

- Yes
- No
- Maybe, not sure

3. According to you, what education programs can improve the understanding and prescribing of complex generics by doctors? *

Mark only one oval per row.

	Strongly disagree	Disagree	Neutral	Agree	Strongly agree
**Workshop initiated by healthcare professionals**	◯	◯	◯	◯	◯
**Training by government authorities**	◯	◯	◯	◯	◯
**Training program supported by companies**	◯	◯	◯	◯	◯
**International based advance training**	◯	◯	◯	◯	◯
**Continuing Medical Education on complex generics**	◯	◯	◯	◯	◯

4. Appropriately in how many patients in a month do you prescribe complex drugs such as liposomal injection such as Doxin- Doxorubicin, Dry powder inhaler Symbicort- Budesonide+Formoterol, Restasis- Generic cyclosporin emulsion, Copaxone- Generic glatiramer acetate? *

Mark only one oval.

- Less than 5 patients
- 5-10 patients
- 10-20 patients
- 20-30 patients
- More than 30 patients

5. What are complex generics according to your perspective? *

Mark only one oval.

- Generic medicine with multiple drugs
- Generic version of drug device combination products
- Generic medicines of advanced drug formulation
- Generic medicines of orphan drug that need additional diagnostic tests before initiating treatment
- Generic version of complex active ingredients, formulations, dosage forms, or routes of administration, or are complex drug-device combination products
- Not sure

6. How confident are you about the quality, safety, and efficacy of complex generics? *

Mark only one oval.

- Extremely not confident
- Not so confident
- Neutral
- Somewhat confident
- Extremely confident

7. What source of information do you rely on to assure safety and quality of complex generics while prescribing? *

Mark only one oval.

- Detailing from medical representative
- Research articles published in scientific journal
- Conferences, workshops and seminars
- Colleagues/peers/Key opinion leaders
- Continuing Medical Education

8. Do you believe complex generics differ from simple generics? *

Mark only one oval.

- Yes
- No
- Not sure

9. How do complex generics differ from simple generics as per your perspective? *

Mark only one oval.

- ( ) Complexities in formulation
- ( ) Advanced delivery routes
- ( ) For rare disease treatment
- ( ) Multiple ingredients
- ( ) Advanced generic product making difficulty in establishing clinical equivalence
- ( ) Not sure

10. Which among the following, according to you, can be termed as complex generics? (Select as many answers as applicable) *

Check all that apply.

- [ ] Nano-technological products
- [ ] Long acting complex injections
- [ ] Drug-device combination products (Inhalers and nasal sprays)
- [ ] Generic version of biotechnological medicine
- [ ] Extended/delayed release tablets

11. To what level does your current job involve practical or hands-on experience with complex generic products? *

Mark only one oval.

- ( ) Not at all involved
- ( ) Slightly involved
- ( ) Moderately involved
- ( ) Highly involved
- ( ) Fully involved

12. Rate how much the following factors affect prescribing a complex generic drug? *

Mark only one oval per row.

	Strongly disagree	Disagree	Neutral	Agree	Strongly agree
**Safety**	◯	◯	◯	◯	◯
**Bioequivalence**	◯	◯	◯	◯	◯
**Manufacturing quality**	◯	◯	◯	◯	◯
**Lack of physician awareness**	◯	◯	◯	◯	◯
**Patient acceptance**	◯	◯	◯	◯	◯

13. How often do you hear of Indian marketed complex generic products in your daily practice? *

Mark only one oval.

- ◯ Never
- ◯ Rarely
- ◯ Occasionally
- ◯ Frequently
- ◯ Very frequently

14. In your opinion, what could be the reason for the marketing of complex generics? *

Mark only one oval per row.

	Strongly disagree	Disagree	Neutral	Agree	Strongly agree
**Patient compliance**	⬭	⬭	⬭	⬭	⬭
**Cost**	⬭	⬭	⬭	⬭	⬭
**Accessibility**	⬭	⬭	⬭	⬭	⬭
**Efficacy**	⬭	⬭	⬭	⬭	⬭
**Quality of medicine**	⬭	⬭	⬭	⬭	⬭

15. Which factors contribute to your prescribing decision for a complex generic drug? Rate their relative importance. *

Mark only one oval per row.

	Extremely unimportant	Somewhat unimportant	Neutral	Somewhat important	Extremely important
**Published clinical data**	⬭	⬭	⬭	⬭	⬭
**CME/workshop information**	⬭	⬭	⬭	⬭	⬭
**Medical representative information**	⬭	⬭	⬭	⬭	⬭
**Regulatory approval**	⬭	⬭	⬭	⬭	⬭
**Hospital formulary/protocols**	⬭	⬭	⬭	⬭	⬭
**Availability in hospital pharmacy**	⬭	⬭	⬭	⬭	⬭
**Affordability to the patient**	⬭	⬭	⬭	⬭	⬭
**Peer/colleague recommendations**	⬭	⬭	⬭	⬭	⬭

16. How important is the cost of complex generics when prescribing them to a patient? *

Mark only one oval.

- ⬭ Extremely not important
- ⬭ Not so important
- ⬭ Neutral
- ⬭ Somewhat important
- ⬭ Extremely important

17. In your opinion, what could be the best value assessment technique for complex generics? *

Mark only one oval.

- Cost-utility analysis
- Cost-consequence analysis
- Cost-benefit analysis
- Cost-minimization analysis
- Cost-effective analysis

18. What factors are important to you while prescribing complex generics? *

Mark only one oval per row.

	Extremely not important	Somewhat not important	Neutral	Somewhat important	Extremely important
**Cost**					
**Clinical study report**					
**Patient compliance**					
**Regularity of medical representative visit**					

19. Do you agree whether complex generics need extra regulatory scrutiny? *

Mark only one oval.

- Strongly disagree
- Disagree
- Neutral
- Agree
- Strongly agree

This content is neither created nor endorsed by Google.

Google Forms
